# E-cigarette Use and Severe Coronavirus Disease 2019 (COVID-19) Outcomes: A Meta-Analysis

**DOI:** 10.7759/cureus.59591

**Published:** 2024-05-03

**Authors:** Karen Valadez-Cuen, Tulsi Bhatt, Ileana E Mendez, Dhanshree Solanki, Nawal Abdi, Vrushali Shelar, Jerry J Akplor, Sai Akhila Reddy Bhumanapalli, Suprada Vinyak, Digantkumar Patel, Raghavendra Tirupathi, Viray Shah, Urvish K Patel, Rishabh K Rana

**Affiliations:** 1 Department of Internal Medicine, Las Palmas Del Sol Healthcare, El Paso, USA; 2 Department of Internal Medicine, Pramukhswami Medical College, Karamsad, IND; 3 Department of Medical Sciences, Universidad Autónoma de Centro América (UACA), San José, CRI; 4 Department of Hospital Administration, Rutgers University, New Brunswick, USA; 5 Department of Internal Medicine, University Hospitals Cleveland Medical Center, Cleveland, USA; 6 Department of Internal Medicine, Saratov State Medical University, Saratov, RUS; 7 Faculty of Medicine, Hebei North University, Zhangjiakou, CHN; 8 Department of Internal Medicine, State University of New York (SUNY) Downstate Health Sciences University, School of Public Health (SPH), New York, USA; 9 Department of Internal Medicine, Wellmont/Norton Community Hospital (NCH), Norton, USA; 10 Department of Medicine, Springfield Memorial Hospital, Springfield, USA; 11 Department of Internal Medicine, Keystone Health, Chambersburg, USA; 12 Department of Hospital Medicine, MedStar Good Samaritan Hospital, Baltimore, USA; 13 Department of Public Health and Neurology, Icahn School of Medicine at Mount Sinai, New York, USA; 14 Department of Preventive and Social Medicine/Community Medicine, Shaheed Nirmal Mahto Medical College and Hospital (Erstwhile Patliputra Medical College), Dhanbad, IND

**Keywords:** sars-cov, sars-cov-2 and covid-19, covid-19, effects of vaping, public health and safety, meta-analysis, e-cigarette smoking

## Abstract

E-cigarettes have been known to cause varied poor health outcomes prior to coronavirus disease 2019 (COVID-19), but after the impact of COVID-19, evidence came out that was, in some instances, not as expected regarding the severity of COVID-19 among e-cigarette users (vapers). A meta-analysis was performed on the available evidence to comprehensively find the effect of COVID-19 on existing or past e-cigarette users (vapers). The Meta-analysis of Observational Studies in Epidemiology (MOOSE) guidelines were used to perform this meta-analysis. PubMed was searched for observational studies that described outcomes after COVID-19 positivity from December 1, 2019, to December 2023. Medical Subject Headings (MeSH) keywords were used for searching the relevant studies highlighting the relationship between COVID-19 and e-cigarette users. Calculations for pooled prevalence, 95% confidence interval (95% CI), weights for current e-cigarette users and vapers, and outcomes (events) were made. To analyze the data, Review Manager V.5.4 was used. The I² statistic was used to assess statistical heterogeneity. The I² statistic of >50% was considered significant heterogeneity. The "leave-one-out" method was used for sensitivity analysis. Out of 3231 studies, four studies reported data on vaping and non-vaping status and composite outcomes, resulting in a sample size of 653 COVID-19-positive cases. The pooled prevalence of being COVID-19 positive, having symptoms, or visiting an emergency room was 7.78% (653/8392). COVID-19 patients with current vaping status had decreased odds of poor outcomes compared to non-smokers, with a pooled odds ratio (OR) of 0.09 (95% CI 0.00-2.42; p>0.05) with heterogeneity between studies (I²=99%, p=0.15). Because of difficulties related to data collection and other factors, this meta-analysis was unable to conclusively establish the correlation between e-cigarette usage and severe COVID-19 outcomes such as hospitalization, admission to the intensive care unit, and fatality. Additional research using more detailed data is necessary to fully understand this correlation.

## Introduction and background

Since 1964, the prevalence of traditional smoking of cigarettes has decreased by 50%, and it is believed national and international restrictive regulations have had their contributions along with the very well-known noxious effect on our overall long-term health. The prevalence of smoking was estimated to decrease below 10% within the next two decades using the SimSmoke tobacco control policy simulation model. However, it was not until Electronic Nicotine Delivery Systems (ENDS) products, electronic nicotine delivery systems, made it to the hands of non-smokers and traditional cigarette smokers that it could affect the trajectory of this statistical analysis [1]. 

ENDS products, such as e-cigarettes or, more colloquially, "vaping," have been noted to help people quit traditional smoking, while to others, they have been the gateway to starting [1,2]. As ENDS products became more widely available, marketing strategies had an impact on their popularity among younger generations. From 2011 to 2017, there was a rapid increase in the use of vaping among adolescents [3]. In 2019, there was an increase of 25% of 12th-grade students who reported its use in the past 30 days [4]. In addition, it was found that there was an increase of 46.2% of e-cigarette users among 18- to 25-year-olds in 2017-2018 (5.2-7.6%) [4]. ENDS products are commonly considered the "healthier alternative" to traditional cigarette smoking, but this common misconception began to change in 2019 when emergency rooms started to receive an unprecedented number of atypical patients with chronic obstructive pulmonary disease (COPD) exacerbation symptoms, in particular young adults with a history of vaping. As this population quickly became recognized, the Centers for Disease Control and Prevention (CDC) categorized this new alarming diagnosis as a diagnosis of exclusion as EVALI: e-cigarette, or vaping, product use-associated lung injury [5].

During the pre-coronavirus disease 2019 (COVID-19) period from April 2019 through February 2020, 2807 EVALI hospitalizations and 68 deaths were reported within the US adolescent and millennial populations [6]. More specifically, EVALI patients mainly consisted of 18-year-old males with a history of e-cigarette use [7].

Investigations were initiated during this time using animal models. One study suggested a possible increase in harmful effects from nicotine use in the form of e-cigarettes owing to an increase in ACE2 receptors in lung tissue, as it was found that this is one of the receptors that COVID-19 binds to [8]. In another study, it was found that e-cigarettes increase the inflammatory response in the lungs without having the appropriate repairing cascade of factors activated specifically by the alpha7 nicotinic acetylcholine receptor (nAChRα7). Additionally, vaping, like traditional smoking, has also been found to increase platelet and neutrophil activity, oxidative stress, and altered endovascular function [9].

The outbreak of COVID-19, the virus causing the global pandemic since March 11, 2020, coincided with the discovery of new cases known as EVALI. In February 2020, the CDC stopped reporting EVALI cases due to the COVID-19 outbreak [10]. After data regarding smokers and COVID-19 was analyzed, conflicting results were published. Some suggested vaping having a protective effect, while others concluded contrary to this or no association, but all alluded to the worsening of the situation in COVID-19 for traditional cigarette users [11,12]. Gradually, data was collected that either showed e-cigarette use had decreased or it had remained the same during the pandemic, while, for traditional smokers, there was a higher rate of quitting, suggested to be due to the fear of the lung complications of COVID-19 [13,14]. As the number of deaths from COVID-19 continues to dip [15], there is no conclusive evidence as to what is the best management. Some theories even suggest nicotine for treatment in COVID-19 [16].

We still don't have conclusive evidence as to what the association is between vapers and COVID-19: vapers are more susceptible to developing COVID-19, vapers have a poor prognosis if testing COVID-19 positive, or vaping is a protective factor against COVID-19. In this study, we seek to find correlations between vapers and non-vapers among COVID-19-infected individuals based on available evidence in the public domain. The potential impact of this project might shed light on the basis of available data on whether vaping has a positive or negative correlation with COVID-19 infection outcomes. 

## Review

Methods

Endpoints

The primary aim of this study is to evaluate the association between the possibility of having COVID-19 disease with symptoms severe enough to cause either a hospital visit or hospitalization in individuals who are e-cigarette users and those who are not e-cigarette users or are not vaping currently. Current e-cigarette users were defined as those who were using e-cigarettes. The majority of these studies evaluated COVID-19 confirmation using the combined results of reverse transcription-polymerase chain reaction (RT-PCR), serology, and symptoms. Our secondary aim was to evaluate the composite poor outcomes associated with COVID-19 hospitalized patients who were currently smoking or using e-cigarettes. The composite poor outcome was defined as intensive care unit (ICU) admission, hospitalization, severe disease mandating an emergency room visit, and in-hospital mortality.

Search Strategy and Selection Criteria

**Figure 1:**
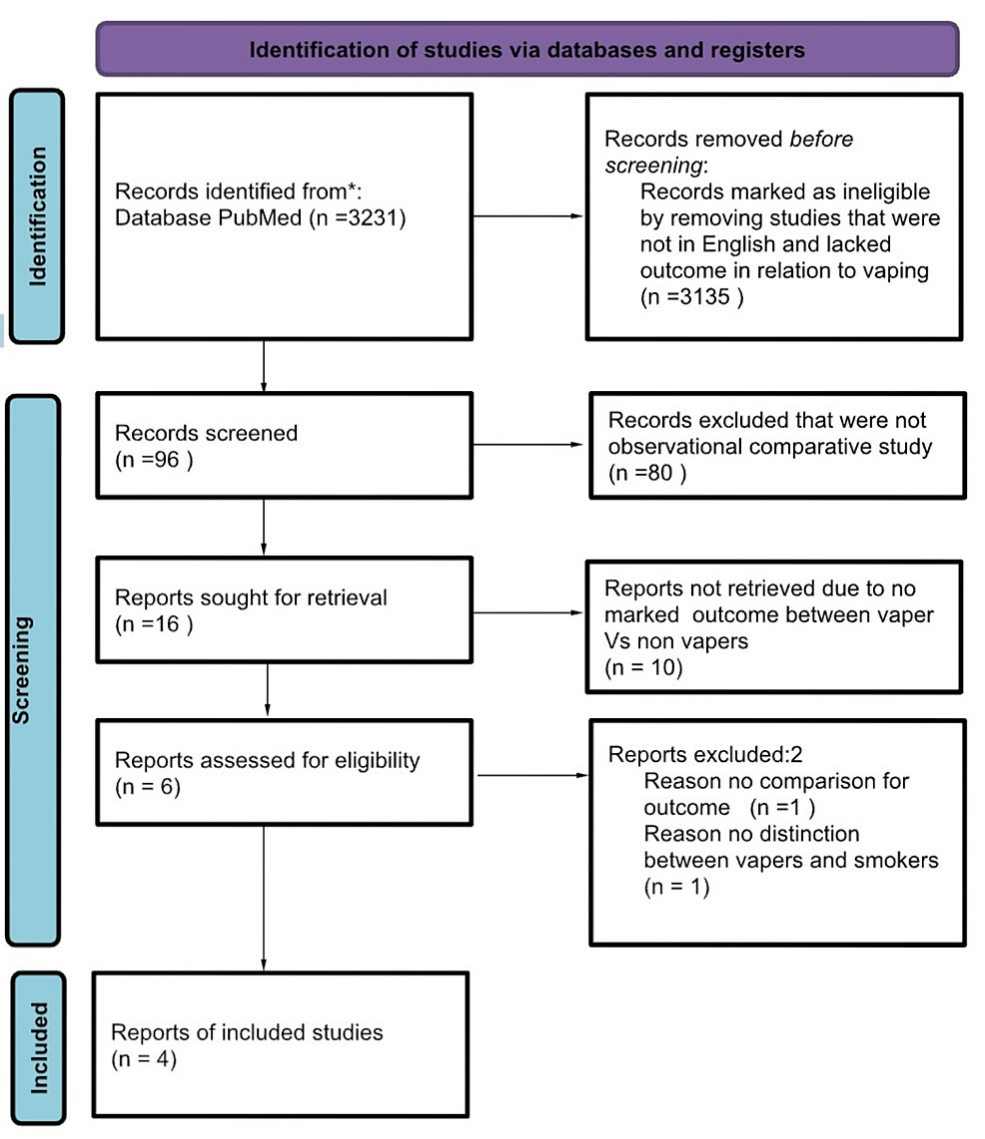
PRISMA flow diagram for selected studies in the meta-analysis PRISMA: Preferred Reporting Items for Systematic Reviews and Meta-Analyses Reference: [18]

The Meta-analysis of Observational Studies in Epidemiology (MOOSE) guidelines were used to perform this meta-analysis [17]. PubMed was searched for observational studies that described outcomes after COVID-19 positivity from December 1, 2019, to December 2023 using the following keyword/Medical Subject Headings (MeSH) terms: ((COVID-19 [Title/Abstract]) OR coronavirus [Title/Abstract]) OR SARS-CoV-2 [Title/Abstract] OR 2019-nCoV [Title/Abstract]. All COVID-19-positive outcome studies were included. Non-observational, non-English, non-full-text, and animal research were excluded. The Preferred Reporting Items for Systematic Reviews and Meta-Analyses (PRISMA) flow diagram of the literature search and study selection process is described in Figure 1.

Study Selection

Abstracts were reviewed, and articles were retrieved if they mentioned current e-cigarette or vaper status. VS and RKR independently screened all identified studies and assessed full texts to decide eligibility. Any disagreement was resolved through discussion with other reviewers (IM and KV).

Data Collection

From the included studies, data relating to patient characteristics, outcomes of interest, and status of vaping and e-cigarette users and comparative data for non-vapers or non-e-cigarette users were given. UP and RKR collected data using prespecified forms, and VS resolved discrepancies. Extracted study characteristics were as follows: publication year, country, sample size, age, e-cigarette user/vaper, non-vaper or non-e-cigarette users, and outcomes. For secondary aim evolution, data of composite outcomes (poor vs. non-poor) were collected.

Assessment of Risk of Bias

The Cochrane Collaboration tool was used to evaluate the quality of the included studies, and the risk of bias was shown using the in-built Review Manager 5.4 risk of bias tool [19].

Statistical Analysis

The primary aim was evaluated using comprehensive meta-analysis software; we calculated pooled prevalence, 95% confidence interval (95% CI), and weights for current e-cigarette users/vapers and outcomes (events). To analyze the data, Review Manager V.5.4 (The Nordic Cochrane Centre, The Cochrane Collaboration, Copenhagen, Denmark) was used. To reduce selection bias, we used data from the most severe outcome if the study had multiple outcome comparisons. Each study used the Mantel-Haenszel formula to calculate dichotomous variables to obtain ORs and 95% CIs to describe the relationship between current e-cigarette users/vapers and COVID-19 patient outcomes. To conservatively estimate ORs and 95% CI, random-effect models were used regardless of heterogeneity to estimate the combined effect and precision. A p-value of <.05 was considered as statistically significant. The I² statistic was used to assess statistical heterogeneity. The I² statistic of >50% was considered significant heterogeneity. The leave-one-out method for sensitivity analysis was performed to assess the effect of publication bias and heterogeneity by excluding outlying studies on the funnel plot. The pooled OR and 95% CI are represented in the form of forest plots. Each square on the chart area represents an individual study, and the area of each square is equivalent to the weight of the study, which is the inverse of the study variance. The diamond represents the summary measures and the width corresponds to the 95% CI.

Results

As of August 30, 2023, four studies were used in this meta-analysis with confirmed COVID-19 cases and giving a status about their vaping status/current e-cigarette user status. Out of these four studies, one study reported about death as an outcome (41/5817 in current vapers vs. 3108/5817 in non-smokers), two studies reported about hospitalization (118/11496 in current vapers vs. 8146/15468 in non-smokers, and two reported about COVID-19 positivity (57/2426 in current vapers vs. 57/3383 in non-smokers), while one reported about ICU admission after COVID-19 (11/1527 in current vapers vs. 93/1527 in non-smokers) [20-23] (Table 1).

**Table 1:** Summary of findings table with details of included studies

Study author	Study period	Study design	Sample size	Mean age	Male (n)	Outcomes n/N in current vapers vs. non-vapers	E-cigarette smokers
Gaiha et al., 2020 [20]	May 2020	Cross-sectional online survey	4351	18.86	1421	COVID-19-related symptoms: 564/2183 vs. 297/2168. COVID-19-positive diagnosis: 51/2183 vs. 18/2168	Ever-use and past 30-day use of cigarettes only, e-cigarettes only
Kale et al., 2021 [21]	April 30, 2020-June 14, 2020	Cross-sectional online survey	2792	64.7	1339	Current vapers 113.75/455 vs. never vaped 457.52/2128	Current vapers (daily and non-daily), never vapers, ex-vapers (stopped vaping)
Gao et al., 2022 [22]	January 24, 2020-April 30, 2020	Cohort study	7869534	48.2	4 111 200	Hospitalization: e-cigarette use 117/14253 vs. never smoked 8133/14253. Admitted to ICU: e-cigarette use 11/1527 vs. never smoked 937/1527. Death: e-cigarette use 41/ 5817 vs. never smoked 3108/5817	A patient as using e-cigarettes if they had a relevant clinical code recorded in their GP record at study entry
McFadden et al., 2022 [23]	March 1, 2020-February 2, 2021	Prospective study	13059	49	6110	COVID-19-positive emergency department visit vapers 6/243 vs. non-users 39/1215. Hospitalization vapers 1/243 vs. non-users 13/1215	Current vapers only

Meta-Analysis 

**Figure 2:**
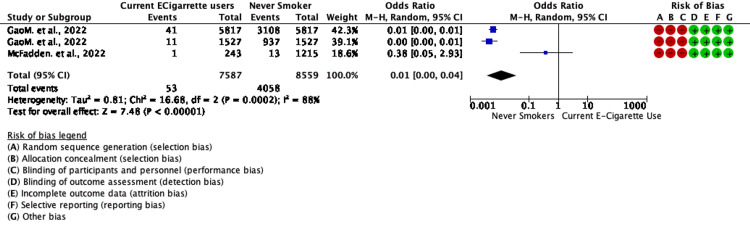
Forest plot for chances of having poor outcomes (emergency admission*, ICU admission, or death) in non-vapers vs. current e-cigarette users in selected studies *emergency admission defined as visit to an emergency department References: [22-23]

**Figure 3:**
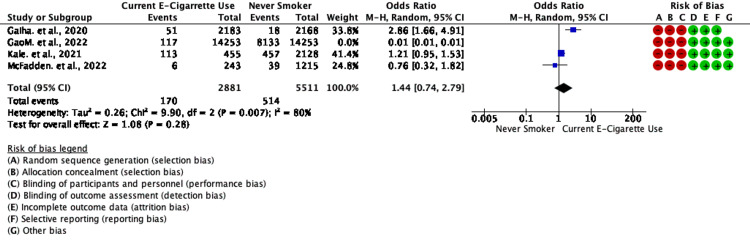
Forest plot for chances of being COVID-19 positive or having emergency room visit as an outcome in non-vapers vs. current e-cigarette users in selected studies. For sensitivity analysis, one study was left out References: [20-23]

A total of four studies reported data on vaping and non-vaping status and composite outcomes giving a total sample size of 684 COVID-19-positive cases or having COVID-19 symptoms; the pooled prevalence of being COVID-19 positive or having COVID-19 symptoms or visit to the emergency department (no hospitalization ) was 8.15% (684/8392). In patients with poor outcomes, the pooled prevalence of poor outcomes like in-house mortality, ICU admission, or hospitalization after ED visit was 25.46% (4111/16146). Meta-analysis of two studies with three outcomes clubbed as severe outcomes showed that COVID-19 patients with current vaping status decreased odds of severe outcomes like mortality , ICU admission or hospitalization post ED visit compared to non-smokers with a pooled OR of 0.01 (95% CI 0.00-0.04; p<0.00 with heterogeneity between studies (I2 =88%;Tau2=0.81; Chi2=16.68; p<0.00). ( Figure 2) Meta-analysis of all four studies showed that COVID-19 patients with current vaping status increased odds of outcomes like being COVID19 positive or ED visits ( no hospitalization ) or Hospitalization only compared to non-smokers with a pooled OR of 1.44 (95% CI 0.74-2.79; p>0.05) with heterogeneity between studies (I2=80%; Tau2=0.26; Chi2=9.90; p=0.007). For sensitivity analysis, one study was left out. (Figure 3).

Discussion 

In this meta-analysis of four studies having 4795 COVID-19 patients, the following outcomes were compared: visit to the emergency room, hospitalization, ICU admission post-COVID-19, and death among current vapers vs. non-smokers. Higher prevalence and odds of contracting COVID-19 among e-cigarette users compared to the non-e-cigarette user or non-smoker group were found. It was also noted that there was an increase in hospitalizations among smokers compared to non-smokers. This study was found to have low external validity likely due to the summation of variables among the studies obtained.

Gaiha et al. found both e-cigarette-only users and dual users of e-cigarettes and traditional cigarettes were at higher risk of contracting COVID-19. Moreover, dual users had a significantly higher risk of COVID-19-related symptoms [20]. In the HEBECO study, they were unable to find an association between vaping and increased diagnosis of COVID-19 [21]. While the large cohort study that took place in England at the peak of the COVID-19 pandemic found current smokers had a lower risk of COVID-19-specific hospitalization, ICU stay, and death but had an increased all-cause mortality when compared to non-smokers, they did not find an association between e-cigarette use and COVID-19 [22]. McFadden et al. found the frequency of hospitalization among COVID-19-positive vapers vs. COVID-19-positive non-vapers to be low, with a non-significant difference between the two groups with similar frequency results for ED visits [23]. Higher possibilities of contracting COVID-19 in vapers and smokers were postulated, but different studies could not settle this claim [8,20]. Possible mechanisms linking COVID-19 with smoking, including vaping, have been established by the ACE2 receptors. Severe acute respiratory syndrome coronavirus 2 (SARS-CoV-2) enters the host through aerosols and binds to the nasal and airway epithelial ACE2 cellular surface protein. Smoking and nicotine increase lung cell ACE2 receptor expression, which may aid SARS-CoV-2 virus binding and internalization [24,25]. The conundrum regarding the conflicting results of the protective effect of smokers on COVID-19 is not new, as shown by Usman et al. where they elaborated on the smoker's paradox by using adjustments; in the same time period, another study also found the smoker's paradox to be present [12]. In our study, similar to Usman et al., our results were not statistically significant to conclusively say, to be in agreement with other studies till date, that vapers might have lower odds of COVID-19 compared to non-smokers. There are elaborate mechanism pathways described by various authors regarding possible pulmonary epithelial damage resulting in the accentuation of COVID-19 from e-cigarette smoke including EVALI [6,8,26]. Tobacco products have been known to cause not just lung disease but also cancer, cardiovascular disease, diabetes, and diseases of the immune system [27]. COVID-19 has been found to cause mood and sleep disorders, memory and verbal deficits, gastrointestinal symptoms such as nausea, vomiting, or diarrhea, kidney disease, diabetes mellitus, musculoskeletal debility and wasting, cardiovascular disease, immune system dysfunction, scarring of the lung, liver injury presenting as steatosis, congestion, or fibrosis, and even dermatological manifestations in the long term [28].

It has been found that e-cigarette use has an increased stroke risk alongside the typical presentation of an EVALI patient, but nicotine has been suggested as a treatment option for severe COVID-19 [29]. Evidence has been found on the mechanism that demonstrates nicotine could actually mitigate the cytokine storm by downregulating the excess release of cytokines, which we know can be detrimental to COVID-19 [30]. It is known that the NF-B factor induces pro-inflammatory cytokines by translocating to the nucleus and activating the tyrosine kinase JAK2/transcription factor STAT3 pathway. To control and downregulate this inflammatory response, in the CAP signaling pathway, macrophage ACh receptor activation prevents NF-B factor translocation. Kloc et al. and others have postulated, on the basis of studies, that nicotine activates the macrophage through the ACh receptor, thus inhibiting the cytokine storm in the lungs. Thus, it is postulated that smoking/vaping COVID-19 patients may avoid acute respiratory distress syndrome (ARDS) due to nicotine use [31,32]. To date, it is unclear what role nicotine has in COVID-19, but as the number of severe cases continues to decrease, it is more necessary to research its mechanism of action along with its various interactions. 

Strengths and limitations

To our knowledge, this is the first meta-analysis based on the occurrence of various possible COVID-19 outcomes comparing the prevalence of current e-cigarette users versus non-smokers. Our findings are not conclusive but reflect the study qualities that need to be rectified. Limitations of the study include its retrospective nature, unknown frequency of smoking, lack of known drug or alcohol use, unknown comorbid conditions, etc. Also, we lack additional information on the severity and duration of symptoms, as well as clinical interventions. Using only one database for the meta-analysis is also one of our limitations while in our statistical model using a random-effects model and sensitivity analysis to explain our study's high heterogeneity. The random model assumes between-study variability, which might lead to wider confidence intervals and potentially mask true effects. While leaving one study out might not be very powerful for detecting publication bias with a small number of studies, larger observational studies specifically designed to assess vaper interaction with COVID-19, controlling for relevant risk variables and comorbidities, are required to corroborate our results.

## Conclusions

Vapers were more likely to have COVID-19. Using e-cigarettes was not helpful in times of COVID-19 and is bound to cause harm. We could not obtain clear results regarding ICU admission and mortality among COVID-19-positive e-cigarette users vs. COVID-19-positive non-smokers, as we raised various issues regarding the study data itself. Due to the nature of the data collection, among other variables, we could not infer any definitive verdict regarding vapers and their COVID-19 outcomes. Vaping is a problem that needs more attention, and all health advisories are directed to stop vaping. Given the extensively known negative health consequences linked to vaping, such as lung harm and increased susceptibility to respiratory diseases, the prospective benefits for COVID-19 that have not been proven yet are not significant enough to outweigh the confirmed risks. Hence, we put forward the following suggestions: For policymakers, implement more stringent rules regarding the sale and assortment of vaping goods, with a particular focus on preventing their adoption among young people. Furthermore, support public health initiatives that prioritize raising awareness about the dangers of vaping, especially in light of the COVID-19 pandemic. Targeting healthcare professionals, advise all patients, especially those with a higher susceptibility to severe COVID-19 problems, to refrain from vaping. Offer support and provide resources to people who are currently utilizing vaping goods to help them quit smoking. For the general public, it is advised to completely abstain from using vaping products. There is no verified information indicating that they provide protection against COVID-19, while they do pose substantial health hazards. Consider seeking smoking cessation treatments if you are currently utilizing vaping products.
